# Mn tolerance in rice is mediated by MTP8.1, a member of the cation diffusion facilitator family

**DOI:** 10.1093/jxb/ert243

**Published:** 2013-08-20

**Authors:** Zonghui Chen, Yumi Fujii, Naoki Yamaji, Sakine Masuda, Yuma Takemoto, Takehiro Kamiya, Yusufujiang Yusuyin, Kozo Iwasaki, Shin-ichiro Kato, Masayoshi Maeshima, Jian Feng Ma, Daisei Ueno

**Affiliations:** ^1^Faculty of Agriculture, Kochi University, Nankoku 783-8502Japan; ^2^Institute of Plant Science and Resources, Okayama University, Kurashiki 710-0046, Japan; ^3^Graduate School of Agricultural and Life Sciences, The University of Tokyo, Tokyo 113-8657, Japan; ^4^Laboratory of Cell Dynamics, Graduate School of Bioagricultural Sciences, Nagoya University, Nagoya 464-8601, Japan

**Keywords:** CDF family, manganese tolerance, *OsMTP8.1*, rice, transporter, vacuole

## Abstract

Manganese (Mn) is an essential micronutrient for plants, but is toxic when present in excess. The rice plant (*Oryza sativa* L.) accumulates high concentrations of Mn in the aerial parts; however, the molecular basis for Mn tolerance is poorly understood. In the present study, genes encoding Mn tolerance were screened for by expressing cDNAs of genes from rice shoots in *Saccharomyces cerevisiae*. A gene encoding a cation diffusion facilitator (CDF) family member, *OsMTP8.1*, was isolated, and its expression was found to enhance Mn accumulation and tolerance in *S. cerevisiae*. In plants, OsMTP8.1 and its transcript were mainly detected in shoots. High or low supply of Mn moderately induced an increase or decrease in the accumulation of OsMTP8.1, respectively. OsMTP8.1 was detected in all cells of leaf blades through immunohistochemistry. OsMTP8.1 fused to green fluorescent protein was localized to the tonoplast. Disruption of *OsMTP8.1* resulted in decreased chlorophyll levels, growth inhibition in the presence of high concentrations of Mn, and decreased accumulation of Mn in shoots and roots. However, there was no difference in the accumulation of other metals, including Zn, Cu, Fe, Mg, Ca, and K. These results suggest that OsMTP8.1 is an Mn-specific transporter that sequesters Mn into vacuoles in rice and is required for Mn tolerance in shoots.

## Introduction

Manganese (Mn) is an essential micronutrient required by all organisms. In plants, Mn plays roles in oxygen generation in a domain of photosystem II and in decomposing superoxide in mitochondria by Mn-containing proteins. Further, Mn is a cofactor that activates ~35 different enzymes (Williams and Pittman 2010; Broadley *et al*., 2012). In spite of its importance in many biochemical processes, Mn can be toxic to plants growing on acidic and/or poorly drained soils with a highly reduced state and thus very high Mn availability. Excessive accumulation of this metal is characterized by the presence of brown spots on mature leaves (Wissemeier and Horst, 1992), interveinal chlorosis, and necrosis and deformation of young leaves (Foy *et al*., 1978, 1988; Horst and Marschner, 1978).

The threshold of Mn toxicity and the tolerance to excess Mn concentrations varies characteristically according to plant species and their cultivars (Foy *et al*., 1988). Rice (*Oryza sativa* L.) is one of the most Mn-tolerant crops, especially flooded or paddy rice. Some rice species accumulate Mn in their leaves at concentrations as high as 5000 µg g^–1^ dry weight (DW) without showing any toxic symptoms, which is remarkably high when compared with the Mn concentration recorded in barley with toxicity (150 µg g^–1^ DW) (Vlamis and Williams, 1964). Mn toxicity is typically caused by oxidation of excess Mn^2+^ to Mn^3+^ in the apoplast, which is in turn a strong oxidizer of proteins and lipids (Fecht-Christoffers *et al*., 2003*a*, *b*). Sasaki *et al*. (2011) reported that a mutant rice strain with high Mn concentrations in the apoplast exhibited severe necrosis in the leaf blades following exposure to high Mn, unlike the wild type. This suggests that maintaining a low Mn concentration in the apoplastic solution is necessary to avoid Mn toxicity. In this study, however, the Mn concentration in the apoplastic fluids was approximately one-tenth that in the symplastic solution of both wild-type and mutant plants, suggesting the presence of an intracellular mechanism to cope with excess Mn that would account for the Mn tolerance of rice.

The sequestration and compartmentalization of Mn in the vacuoles, endoplasmic reticulum (ER), or Golgi plays crucial roles in Mn tolerance (Williams and Pittman, 2010). A variety of transporters belonging to the families CAX (cation exchanger) (Hirschi *et al*., 2000; Shigaki *et al*., 2003; Korenkov *et al*., 2007; Edmond *et al*., 2009), CDF (cation diffusion facilitator) (Delhaize *et al*., 2007; Peiter *et al*., 2007), and P_2A_-type ATPase (Wu *et al*., 2002; Li *et al*., 2008; Mills *et al*., 2008) mediate these processes, particularly in *Arabidopsis thaliana*. OsCAX1a and OsCAX3 derived from rice confer Mn tolerance on yeast cells (Kamiya *et al*., 2005). Although OsCAX1a resides in the vacuolar membrane (Kamiya *et al*., 2006), the role of these transporters in plants is not clear. Recently, Zhang *et al*. (2012) reported that OsVIT1 and OsVIT2—orthologues of the yeast Fe/Mn transporter CCC1—transport Mn as well as Fe and Zn into vacuoles in yeast, although evidence suggests that they act as vacuolar transporters for Fe and Zn in plants. Thus, the molecular mechanism underlying tolerance to high concentrations of Mn is still poorly understood in rice.

Members of the CDF family, first described by Nies and Silver (1995), are present in numerous organisms, including bacteria, fungi, animals, and plants (Mäser *et al*., 2001), and act as transporters for the divalent cations Zn, Fe, Co, Cd, and Mn (Haney *et al*., 2005; Gustin *et al*., 2011). In plants, CDF proteins are designated as metal tolerance proteins (MTPs). These proteins are classified into three major groups—Zn-CDF, Fe/Zn-CDF, and Mn-CDF—according to their respective major metal substrate (Montanini *et al*., 2007; Gustin *et al*., 2011). Compared with other groups, the Zn-CDF group is well characterized. Van der Zaal *et al*. (1999) designated the CDF protein discovered first as ZAT (zinc transporter of *A. thaliana*), which was later renamed AtMTP1 by Mäser *et al*. (2001). AtMTP1 localizes in vacuolar membranes, and a mutant (*mtp1*) was found to show enhanced sensitivity to high Zn concentrations (Kobae *et al*., 2004). AtMTP1 displayed Zn transport activity in proteoliposomes containing the purified reconstituted AtMTP1 (Bloß *et al*., 2002). These results suggest that AtMTP1 maintains Zn homeostasis by sequestering excess Zn from the cytoplasm into vacuoles. AtMTP3 also mediates vacuolar sequestration of Zn in roots (Arrivault *et al*., 2006). Genes encoding MTPs involved in Zn tolerance and homeostasis are also present in several plant species such as *Medicago truncatula* (*MtMTP1*) (Chen *et al*., 2009), rice (*OsMTP1*) (Yuan *et al*., 2011), *Arabidopsis halleri* (*AhMTP1*) (Dräger *et al*., 2004), and *Thlaspi goesingense* (*TgMTP1*) (Kim *et al*., 2004). Compared with Zn-CDFs, knowledge of the roles of Mn-CDFs is limited. The Mn-CDF group comprises two distinct subgroups termed Groups 8 and 9 (Gustin *et al*., 2011). *Arabidopsis thaliana* harbours four members of the Mn-CDF group, and AtMTP8 is included in Group 8, while AtMTP9/10/11 are members of Group 9. Among these, only the function of AtMTP11 is known. AtMTP11 localizes to the pre-vacuolar compartment or the Golgi network, and it is involved in maintaining Mn homeostasis (Delhaize *et al*., 2007; Peiter *et al*., 2007). Expression of *AtMTP11* in a Mn-sensitive mutant yeast strain restored Mn tolerance to wild-type levels, and the microsomes in the mutants showed enhanced activity for Mn transport. Mutants of *atmtp11* exhibit Mn sensitivity and accumulate higher levels of Mn in shoots and roots than the wild-type plants with the basal supply level of Mn; however, when Mn supply is high, there is no difference in Mn accumulation between the mutant and the wild type. In rice, there are five members of the Mn-CDF group (Gustin *et al*., 2011), and OsMTP8/8.1 and OsMTP9/11/11.1 are classified into Groups 8 and 9, respectively, although their functions are unknown. Other than the Mn-CDF members from *A. thaliana* and rice, ShMTP8 (Group 8) isolated from the Mn-tolerant legume *Stylosanthes hamata* localizes to the tonoplast and confers Mn tolerance when ectopically expressed in *A. thaliana* (Delhaize *et al*., 2003).

To identify genes involved in Mn detoxification in rice, in the present study, a cDNA expression library of genes from rice shoots was constructed and genes that conferred Mn tolerance on *S. cerevisiae* were screened for. Using this approach, a gene, *OsMTP8.1*, encoding a Mn-CDF that confers Mn tolerance presumably by sequestering Mn into vacuoles in rice shoots, was identified.

## Materials and methods

### Plant materials and growth condition

Wild-type rice (*Oryza sativa* L. cv. Nipponbare) and its Tos-17 insertion mutant of *OsMTP8.1* (NF9003) or a line expressing an *OsMTP8.1* small interfering RNA (siRNA) were used in this study. Tos-17 insertion was recognized in exon 6 of the *OsMTP8.1* coding region in the mutant allele (Supplementary Fig. S1A, B available at *JXB* online). Seeds were germinated in tap water for 3 d at 30 °C in the dark after surface sterilization with 0.5% (v/v) NaClO for 1h. After germination, seedlings were transferred to a net floated on a 0.5mM CaCl_2_ solution for 5 d and then on a half-strength Kimura B nutrient solution (pH 5.4) containing the macronutrients MgSO_4_ (0.28mM), (NH_4_)_2_SO_4_ (0.18mM), Ca(NO_3_)_2_ (0.18mM), KNO_3_ (0.09mM), and KH_2_PO_4_ (0.09mM); and the micronutrients Fe(II)SO_4_ (10 µM) or Fe(III)-EDTA (20 µM), H_3_BO_3_ (3 µM), MnCl_2_ (0.5 µM), CuSO_4_ (0.2 µM), ZnSO_4_ (0.4 µM), and (NH_4_)_6_Mo_7_O_24_ (1 µM). The solutions were replenished every 2 d. Transgenic plants were first cultured on gels containing Murashige and Skoog salt mixture (Nippon Seiyaku, Tokyo) for ~100 d after introduction of each plasmid (Hiei *et al*., 1994). The seedlings were cultured in a growth chamber (30 °C, 14h light/25 °C, 10h dark). Twelve-day-old seedlings were exposed to nutrient solutions containing varying concentrations of MnCl_2_ (0.05, 0.5, 200, 500, and 1000 µM) for 15 d. To investigate the effect of inhibiting the expression of *OsMTP8.1* on the accumulation of Mn and other microelements, seedlings of the RNA interference (RNAi) lines were first cultured together with wild-type rice for 11 d and then were exposed to a solution containing 200 µM MnCl_2_ for 10 d. In this experiment, Fe(III)-EDTA was used instead of FeSO_4_ to avoid absorption and deposition of a large amount of Fe in the root apoplast and to determine the concentration of cellular Fe. After Mn treatment, the shoots and roots were harvested and washed twice with deionized water, dried at 70 °C for 2 d, weighed, and analysed for Mn and other metals. For determining the chlorophyll content, the youngest (fourth) and the second youngest (third) leaf blades were harvested, weighed, and used directly for chlorophyll extraction.

### Construction of a rice cDNA expression library and screening yeasts for a rice gene encoding Mn tolerance

To construct a cDNA library for screening, the yeast expr ession vector, pKT10-mycN(1) (Tanaka *et al*., 1990), was modified as follows: pKT10-mycN(1) was amplified using the primers 5′-ATATGGCGGCCGCTGATTGATTGACGACTTGGTTG AACACGTTG-3′ and 5′-GGGAGATAAGTCGACGAATTC CAGATCTTCTTCGG-3′ (underlining indicates the *Not*I and *Sal*I sites, respectively), and then the PCR fragment was self-ligated, yielding pKT10NSmyc. Total RNA was extracted from rice shoots using TRIzol RNA isolation reagents (Life Technologies), and mRNA was purified from total RNA using an Oligotex(dT) spin column mRNA purification kit (Takara). The cDNA library was generated from mRNA using a cDNA library construction kit (Takara) and cloned into the *Not*I and *Sal*I sites of pTK10NSmyc. The Mn-hypersensitive mutant strain Δ*pmr1* (Mat a; his3Δ1; leu2Δ0; met15Δ0; ura3Δ0; PMR1::kanMX4) of yeast (*S. cerevisiae*) defective in Mn transport in the Golgi (Lapinskas *et al*., 1995), and its parental wild-type BY4741 (Mat a; his3Δ1; leu2Δ0; met15Δ0; ura3Δ0) were purchased from Euroscarf (http://web.uni-frankfurt.de/fb15/mikro/euroscarf/index.html; last accessed 22 July, 2013). The cDNA library was introduced into Δ*pmr1*, and the transformants were grown at 30 °C on plates containing a synthetic complete medium consisting of yeast nitrogen base (Difco), amino acids without uracil, and 2% glucose (SC-U/Glu, pH 5.0). After 2 d, colonies were suspended in sterilized water. For primary screening, the yeast suspension was streaked on SC-U/Glu plates containing a toxic concentration of Mn (5mM). Colonies that grew vigorously were selected and rescreened on another plate containing a toxic concentration of Mn. The nucleotide sequence of each cDNA from each colony was determined after amplifying the bacterial DNA using the primers 5′-GAATTACCATGGAGCAGAACTGA-3′ and 5′-GATTTAAAGTAAATTCACTTAAGCCTT-3′ derived from pKT10NSmyc.

### Cloning of a cDNA encoding OsMTP8.1

Total RNA was extracted from rice shoots with the RNeasy Plant Mini kit (Qiagen) and converted to cDNA using SuperScript II reverse transcriptase (Life Technologies) after DNase I (Life Technologies) treatment. The full-length cDNA containing an entire open readiing frame (ORF) of *OsMTP8.1* was amplified by PCR using the primers 5′-AGAAAGGAGAGAGGTGATTCGAT-3′ and 5′-CTAATTCGTTTCACGGTGGAAT-3′, which were designed according to the sequence information of Os03g0226400 deposited in the Rice Annotation Project Database (http://rapdb.dna.affrc.go.jp/; last accessed 22 July 2013). The PCR fragment was subcloned into the pGEM-T Easy vector (Promega) and sequenced using a Big-Dye sequencing kit (Applied Biosystems) on an Applied Biosystems 3130 Genetic Analyzer (Applied Biosystems).

### Functional analysis in yeast

*OsMTP8.1* cDNA was amplified from pGEM-T Easy-*OsMTP8.1* plasmid DNA using the primers 5′-AGAATTCAACAATGGAGG CGAAG-3′ and 5′-TCTCGAGTCATGGTTGGCTGCTA-3′. The product was subcloned into the *Eco*RI and *Xho*I sites of a yeast expression vector pYES2 (Life Technologies). *OsMTP8.1:GFP* cDNA was amplified from pUC18-*OsMTP8.1:GFP* plasmid DNA constructed as described below using the primers 5′-AGAATTCA ACAATGGAGGCGAAG-3′ and 5′-ACCTAGGTTACTTGTACA GCTCGTC-3′. The product was subcloned into the *Eco*RI and *Xba*I sites of pYES2. For the complementation assay, Δ*pmr1* and the Zn-hypersensitive mutant strain Δ*zrc1cot1* (MATa; his3Δ1; leu2Δ0; met15Δ0; ura3Δ0; ZRC1::natMX COT1::kanMX4) (Dräger *et al*., 2004) were transformed with pYES2-*OsMTP8.1*, pYES2-*OsMTP8.1:GFP*, or pYES2, and were grown up to the stationary phase (12h) in liquid medium (SC-U/Glu) followed by incubation for 5h to achieve log-phase growth (OD_600_ ~0.8). The cells were harvested by centrifugation, washed, and resuspended to OD_600_ 2.0 with sterile water. Four 10-fold serial dilutions were prepared in sterile water for each culture, and 5 µl of each dilution was spotted onto SC-U plates containing 2% galactose (SC-U/Gal) with or without 5mM MnCl_2_, 0.25 mM ZnSO_4_, or 0.4 mM CoCl_2_, respectively.

To measure Mn accumulation, the transformed wild-type strain was grown to log phase in SC-U/Glu medium. The cells were washed with sterile water, transferred to SC-U/Gal medium, and the OD_600_ was adjusted with medium to 0.1. After 3h, MnCl_2_ was added to a final concentration of 5mM, and the culture was incubated for 24h. The cells were washed three times with 10mM cooled EDTA (4 °C, pH 5.0) and then dried at 70 °C for 12h. The metal concentrations of the dried samples were determined as described below.

### Real-time reverse transcription–ploymerase chain reaction (RT–PCR) analysis

To investigate the expression pattern of *OsMTP8.1*, shoots and roots of rice were exposed to 0.05, 0.5, and 200 µM MnSO_4_ for 6 d and then total RNA was extracted using an RNeasy Plant Mini kit. Expression levels were analysed using a Thunderbird SYBR qPCR mix (Toyobo) with the primers 5′-AAGGAGGCACATGCTATTGG-3′ and 5′-ATGTTGTGCTCTGGCTTGTG-3′ on a Prism 7300 Real-time PCR System (Applied Biosystems). The expression of *OsNramp5* in the roots was determined using the primers 5′-CAGCAGC AGTAAGAGCAAGATG-3′ and 5′-GTGCTCAGGAAGTAC ATGTTGAT-3′. *Histone H3* was used as an internal standard with the primers 5′-GGTCAACTTGTTGATTCCCCTCT-3′ and 5′-AACCGCAAAATCCAAAGAACG-3′.

### Western blot analysis

The synthetic peptide CDHKPEHNILSKLPSSQP (positions 380–397 of OsMTP8.1), synthesized and conjugated to a carrier protein by Sigma-Aldrich, was used to immunize rabbits to raise polyclonal antibodies against OsMTP8.1. The antiserum was purified through a peptide affinity column. A 15g aliquot of shoots harvested from wild-type or *osmtp8.1* mutant plants was used to prepare microsomes on ice or at 4 °C according to Sugiyama *et al*. (2007). The microsomes were then fractionated using discontinuous sucrose gradients (20–60%). To confirm the subcellular localization of OsMTP8.1, microsomal membranes were extracted in the presence of 2mM MgCl_2_ and were fractionated using a continuous sucrose density gradient, as per the procedure of Mitani *et al*. (2011).

Equal amounts of samples mixed with the same volume of sample buffer containing 100mM TRIS-HCl (pH 6.8), 4% (w/v) SDS, 20% (w/v) glycerol, 0.008% (w/v) bromophenol blue, and 0.12mM dithiothreitol (DTT) were incubated at 65 °C for 10min, and SDS–PAGE was performed using 5–20% gradient gels (ATTO). Proteins were transferred to a polyvinylidene difluoride (PVDF) membrane using an AE-6685 blotting apparatus (ATTO) following the manufacturer’s protocol, and the membrane was incubated with the anti-OsMTP8.1 antibody described above. Anti-Bip (Cosmo bio), anti-V-ATPase (Agrisera), and anti-H^+^-ATPase (Agrisera) polyclonal antibodies against *A. thaliana* proteins were used for detecting the ER, tonoplast, and plasma membrane, respectively. ECL-peroxidase-labelled anti-rabbit antibody (GE Healthcare) was used as a secondary antibody, and an ECL Prime Western Blotting Detection System (GE Healthcare) was used for detecting chemiluminescence of the antigen–antibody complexes. The signal intensities of OsMTP8.1 relative to V-ATPase were calculated using the ImageJ program (version 1.47t, http://rsb.info.nih.gov/ij/index.html; last accessed July 22, 2013).

### Immunohistochemical detection of GFP expression under the OsMTP8.1 promoter

To construct an expression vector to assess the tissue specificity of the *OsMTP8.1* promoter, a 2954bp region upstream of the translational start codon of *OsMTP8.1* was amplified from Nipponbare genomic DNA using the primers 5′-AGGTACCTG TGCATGGAGTGTGCAAGA-3′ and 5′- AGTCGACGAATCAC CTCTCTCCTTTCT-3′. The putative promoter region was cloned into the *Kpn*I and *Sal*I sites of the binary vector pPZP2H-lac (Fuse *et al*., 2001) carrying a nopaline synthase (NOS) terminator. The sequence encoding green fluorescent protein (GFP) was amplified using the primers 5′-AGTCGACATGGTGAGCAAGGGCGA-3′ and 5′-AACTAGTTACTTGTACAGCTCGTCC-3′ and cloned into the *Sal*I and *Spe*I sites of pPZP2H-*pOsMTP8.1*. The construct was introduced into rice calluses derived from Nipponbare using *Agrobacterium*-mediated transformation (Hiei *et al*., 1994).

To detect GFP in the transgenic rice leaves, an antibody against GFP (A11122; Molecular Probes) was used. Leaf blades of *pOsMTP8.1:GFP* transgenic and wild-type rice (negative control) were used for immunostaining of GFP as described previously (Yamaji and Ma, 2007). The fluorescence of the secondary antibody (Alexa Fluor 555 goat anti-rabbit IgG; Molecular Probes) was observed using a confocal laser scanning microscopy (LSM700; Carl Zeiss).

### Transient expression of an OsMTP8.1–GFP fusion protein

An *OsMTP8.1* cDNA fragment without a translational stop codon was amplified from the full-length *OsMTP8.1* cDNA using the primers 5′-ACTCGAGATGGAGGCGAAGG-3′ and 5′-TCCATGGATGGTTGGCTGCTAG-3′, and was cloned into the *Sal*I and *Nco*I sites of pUC18 carrying the *Cauliflower mosaic virus* (CaMV) 35S promoter and NOS terminator. Gold particles (1 µm) coated with the either the *OsMTP8.1:GFP* or *GFP* constructs were delivered into onion epidermal cells using particle bombardment (PDS-1000/He particle delivery system, Bio-Rad) using 1100 psi pressure disks. Twelve hours later, GFP fluorescence was observed using confocal laser microscopy (LSM700; Carl Zeiss).

### Generation of RNAi transgenic plants

To prepare a hairpin RNAi construct, a 270bp fragment of *OsMTP8.1* cDNA (217bp to 486bp from the transcriptional start) was cloned as inverted repeats into the pANDA vector under the control of the maize ubiquitin promoter (Miki and Shimamoto, 2004). The primers used for amplifying the 270bp fragment were 5′-AAAAAGCAGGCTTCAGATCCTTTGAG GAAGTTGATT-3′ and 5′-AGAAAGCTGGGTT-AGGATACT TGTAGACGTTGATG-3′. Transgenic lines were generated according to Hiei *et al*. (1994). The expression levels of *OsMTP8.1* in the RNAi lines were estimated using semi-quantitative RT–PCR and the primer sets described in ‘Real time RT–PCR analysis’ above.

### Mn uptake

To compare Mn uptake between knockout and wild-type plants, 22-day-old seedlings were exposed to 50% Kimura B nutrient solution containing 200 µM Mn. After 24h, two seedlings were transferred to a test tube (16ml, 1.5cm diameter×5cm length) containing 14ml of the solution above but buffered at pH 5.4 with 1mM 2-(*N*-morpholino)-ethanesulphonic acid (uptake solution). The seedlings were incubated for 16h from 23:00h to 15:00h (25 °C, 7h dark/30 °C, 9h light). The weights of the tubes with the uptake solution and seedlings were recorded before and after exposure to determine the loss of water due to transpiration. Roots were then harvested, dried at 70 °C, and weighed. Mn concentrations of the uptake solutions were determined as described below. The value obtained for the blank was subtracted from all measurements.

### Determination of metal concentration

Dried samples were digested with concentrated HNO_3_ (60%) at 140 °C for plants tissues and 95 °C for yeast. The metal concentrations in the digests were determined using atomic absorption spectrometry (AA-6800, Shimadzu).

### Determination of chlorophyll concentration

Fresh leaf blades were ground in 10ml of 80% acetone with quartz sand and Na_2_CO_3_. The total chlorophyll content of the extracts was determined using the method of Arnon (1949).

### Nucleotide sequence analysis

Sequence alignments were generated using ClustalW (http://clustalw.ddbj.nig.ac.jp/; last accessed 22 July 2013). Protein transmembrane domains were predicted using the HMMTOP program (version 2.0, http://www.enzim.hu/hmmtop/; last accessed on 22 July, 2013). Amino acid sequence similarities were analysed using the NPS@ Web server (http://npsa-pbil.ibcp.fr/cgi-bin/npsa_automat.pl?page=/NPSA/npsa_clustalw.html; last accessed 22 July 2013). The phylogenetic tree was constructed using MEGA 5 software (version 5.05, released from http://www.megasoftware.net/; last accessed on July 22, 2013) after ClustalW alignment.

## Results

### Isolation of rice cDNAs conferring Mn tolerance upon S. cerevisiae

**Fig. 1.**
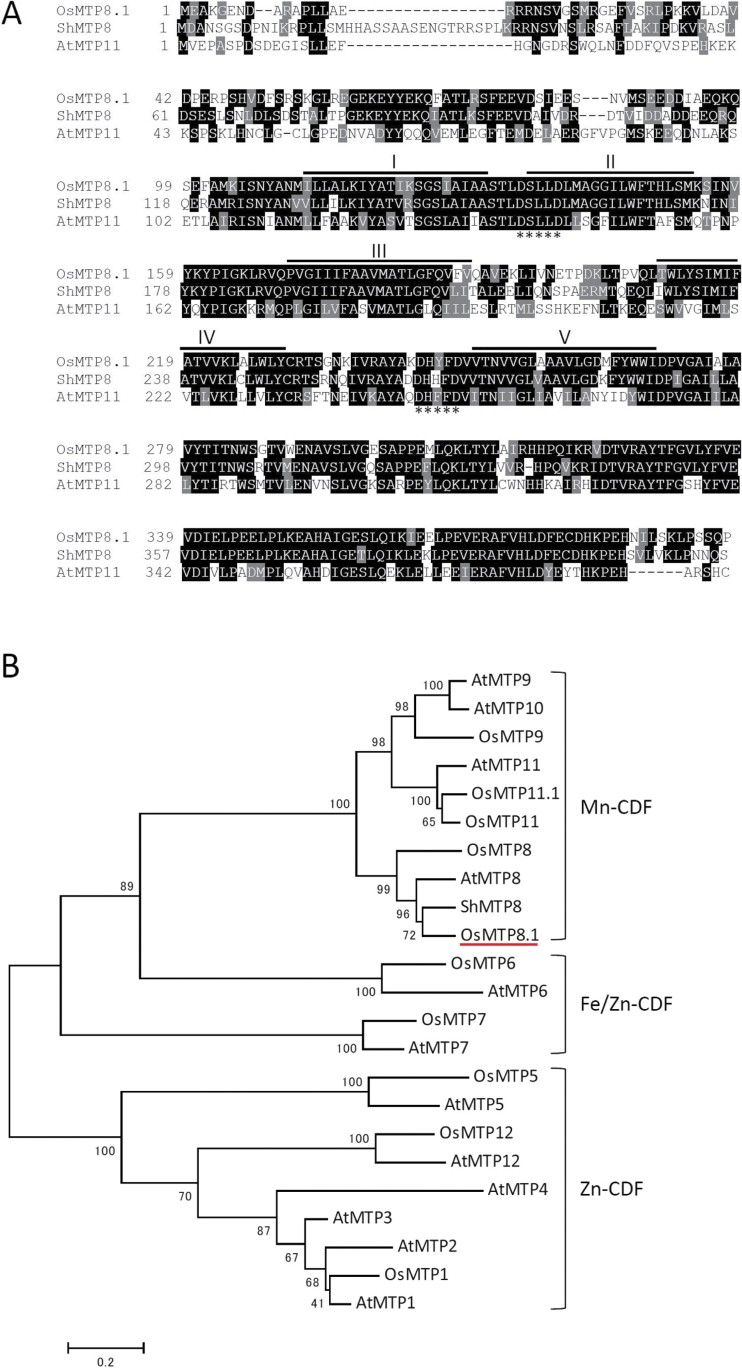
Sequence analysis. (A) ClustalW alignment of the CDF proteins from rice (OsMTP8.1), *Stylosanthes hamata* (ShMTP8), and *Arabidopsis thaliana* (AtMTP11). Shading indicates identical (black) or similar (grey) amino acid residues. The transmembrane domains of OsMTP8.1 predicted by the HMMTOP program (version 2.0) are shown as lines above the sequence. Asterisks indicate the conserved sequence (DxxxD) in members of the Mn-CDF group (Montanini *et al*., 2007). (B) Phylogenetic tree of the CDF family sequences generated using MEGA5 software (ver. 5.05).

To identify genes involved in mediating Mn tolerance in rice shoots, expression of a cDNA was used to identify rice genes that conferred Mn tolerance on *S. cerevisiae*. The cDNA library prepared from rice shoots was introduced into the *pmr1* mutant (Δ*pmr1*) strain of *S. cerevisiae* that is hypersensitive to Mn. *Saccharomyces cerevisiae* colonies that could grow on agar medium containing a toxic concentration of Mn (5mM) were selected. Forty colonies were selected that harboured either one of three different sizes of cDNA fragments that corresponded to Os03g0226400, Os12g0188700, or Os06g0531900 in The Rice Annotation Project Database (http://rapdb.dna.affrc.go.jp/). The predicted Os12g0188700 and Os06g0531900 polypeptides are similar to thioredoxin and lipase, respectively; however, these functions have not been experimentally confirmed. A cDNA that mapped to rice chromosome 3 (Os03g0226400) encodes a member of the CDF family of transporters designated OsMTP8.1 by Gustin *et al*. (2011). The OsMTP8.1 sequence comprises 398 amino acids residues (44.7kDa), and it is predicted to contain five transmembrane domains (TMDs) (Fig. 1A). Based on a multiple sequence alignment and the substrate specificities of certain characterized transporters, the CDF family members are classified into three major groups as follows: Zn-CDF, Fe/Zn-CDF, and Mn-CDF (Fig. 1B; Montanini *et al*., 2007). Mn-CDF sequences can be differentiated by the consensus sequence DxxxD (x=any amino acid) in TMDs II and V (Montanini *et al*., 2007). These residues are present in OsMTP8.1 as well as in the Mn transporters AtMTP11 (GenBank locus AEC09679) and ShMTP8 (originally ShMTP1) (GenBank locus AAO38707) (Delhaize *et al*., 2003, 2007; Peiter *et al*. 2007). The predicted sequence of OsMTP8.1 is 48.0% and 70.4% identical to those of AtMTP11 and ShMTP8, respectively.

**Fig. 2.**
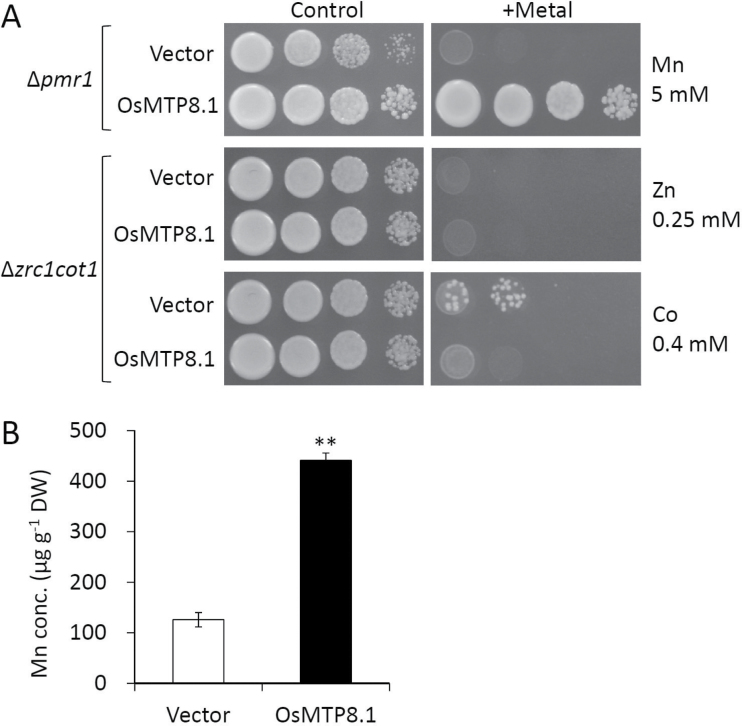
Effect of *OsMTP8.1* expression on tolerance to Mn, Zn, and Co and accumulation of Mn in *Saccharomyces cerevisiae.* (A) Yeast complementation assay. The yeast mutants Δ*pmr1* or Δ*zrc1cot1* carrying the pYES2 empty vector or pYES2-*OsMTP8.1* were used. A 5 µl aliquot (OD_600_=2.0) of serial dilutions (10-fold) was spotted onto SC-U/Gal medium with or without (control) supplementation with 5mM MnCl_2_, 0.25mM ZnSO_4_, or 0.4mM CoCl_2_. Plates were incubated for 48h at 30 °C in the dark. (B) Mn accumulation by the *S. cerevisiae* strain BY4741. BY4741 was transformed by the pYES2 empty vector or pYES2-*OsMTP8.1* was cultured in liquid SC-U/Gal medium supplemented with 5mM MnCl_2_ at an initial OD_600_=0.1 for 24h. Data represent means ±SD (*n*=3). Significant differences between the empty vector and OsMTP8.1 calculated using Student’s *t*-test are indicated by ** (*P* < 0.01).

To characterize OsMTP8.1 functionally, it was first determined whether its expression affected the Mn sensitivity of Δ*pmr1*. Cultures of Δ*pmr1* carrying either the pYES2 empty vector or pYES2-*OsMTP8.1* grew similarly in a medium containing the normal (non-toxic) Mn concentration (Fig. 2A). The growth of Δ*pmr1* transformed by the empty vector was inhibited in the presence of a high Mn concentration, in contrast to cells harbouring the *OsMTP8.1* expression vector. On the other hand, *OsMTP8.1* expression did not restore sensitivities to Zn or Co in Δ*zrc1cot1*. To investigate further the metal transport activity and selectivity of OsMTP8.1, the accumulation of Mn and other metal cations was compared in wild-type strains that expressed *OsMTP8.1* or that did not express *OsMTP8.1*. Analysis of cells growing in liquid medium with 5mM Mn, a concentration that did not restrict cell growth, showed that cells expressing *OsMTP8.1* accumulated 3.5-fold the amount of Mn accumulated in the vector control after 24h (*P* < 0.01, Fig. 2B). Significant differences were not detected in the concentrations of other metals in the control or *OsMTP8.1*-expressing cells (data not shown).

### Expression pattern and localization of *OsMTP8.1*

**Fig. 3.**
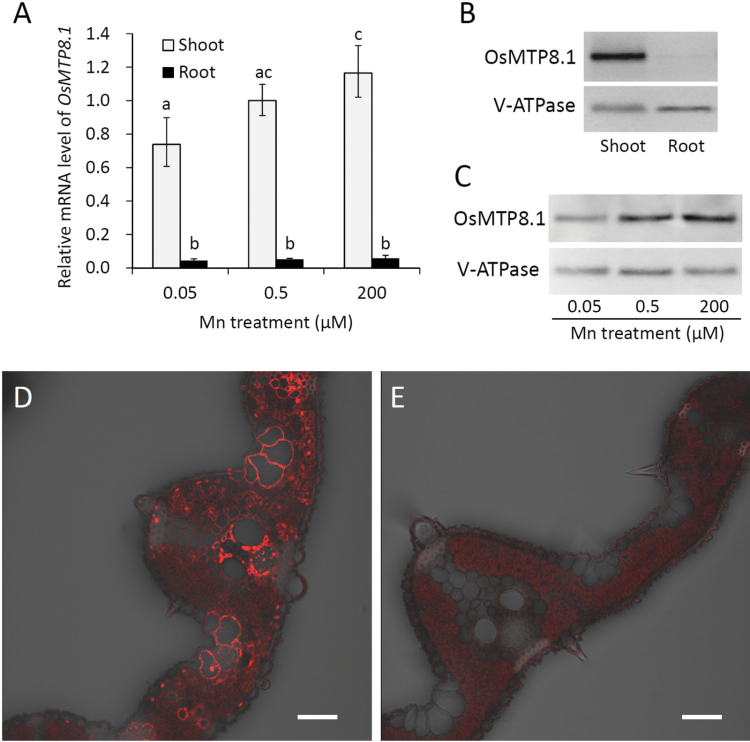
Expression pattern of OsMTP8.1. (A) Quantitative real-time RT–PCR analysis of *OsMTP8.1* expression in rice shoots and roots grown in different Mn concentrations. Plants were hydroponically grown for 12 d and then for 6 d in a solution containing varying concentrations of Mn (0.05, 0.5, and 200 µM). *Histone H3* was used as an internal control. Expression relative to the shoots in the presence of 0.5 µM Mn is shown. Data represent the mean ±SD (*n*=3). Different letters indicate a significant difference at *P* < 0.05 using Tukey’s test. (B) Western blot analysis of OsMTP8.1 in shoots and roots. The tonoplast marker protein V-ATPase was detected using a specific antibody. (C) Western blot analysis of OsMTP8.1 in shoots grown in the presence of varying concentrations of Mn (0.05, 0.5, and 200 µM). (D, E) Immunostaining of the leaf blades of the *OsMTP8.1* promoter–*GFP* transgenic line (D) and wild-type rice (E). Scale bars=50 µm.

To investigate the tissue-specific expression pattern and response to Mn, mRNA levels, determined using quantitative real-time RT–PCR, of both shoots and roots were compared in the presence of different Mn concentrations as follows: adequate (0.5 µM), reduced (0.05 µM), and excess (200 µM). *OsMTP8.1* was mainly expressed in the shoots under all conditions, and its expression level was slightly higher in the presence of excess Mn compared with the reduced level (*P* < 0.05, Fig. 3A). The level of OsMTP8.1 accumulation was also examined using western blot analysis. The specificity of the anti-OsMTP8.1 antibody is shown in Supplementary Fig. S1C at *JXB* online. A band with the expected size (44.7kDa) was detected; however, two additional bands—although the signal from the larger band was weak—were also detected in the 75–100kDa region in the wild-type. Because the members of CDF family transporters function as dimers or oligomers (Blaudez *et al*., 2003), the additional bands were probably derived from dimers or oligomers that were not dissociated under the denaturing and reducing conditions used here. Alternatively, these additional bands might represent translated products from alternatively spliced mRNAs of *OsMTP8.1*, although no such transcripts have been reported in plant *CDF*. Because all the bands were detected at much lower levels in extracts from the mutant than in the extracts from the wild type, it was concluded that the antibody might specifically detect OsMTP8.1. In the present study, however, the degree of dissociation could not be controlled; therefore, data are presented pertaining to the bands of the expected size, unless otherwise mentioned. OsMTP8.1 was predominantly detected in aerial tissues (band corresponding to the middle size) (Fig. 3B), which was consistent with the mRNA expression data. Further, the protein levels increased to 140% or decreased to 86% by excess or reduced levels of Mn, respectively (Fig. 3C).

To investigate the tissue specificity of *OsMTP8.1* expression, a transgenic plant carrying the *OsMTP8.1* promoter fused to *GFP* was generated. Immunostaining with an anti-GFP antibody showed that GFP was detected in all cells of the leaf blade, and its levels were particularly high in the parenchyma cells of the xylem and phloem (Fig. 3D). No signal other than the intrinsic fluorescence of chloroplasts was detected in wild-type plants (Fig. 3E), indicating the specificity of the antibody.

**Fig. 4.**
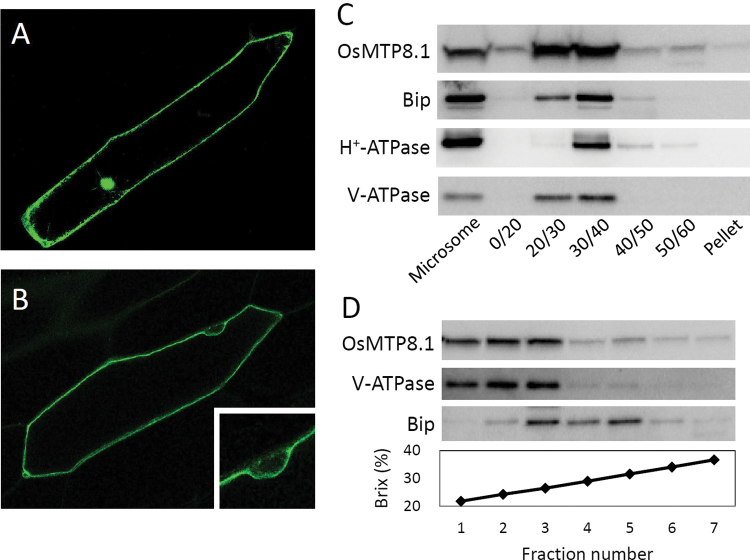
Subcellular localization of OsMTP8.1. (A, B) Localization of GFP (A) and the OsMTP8.1:GFP fusion protein (B) transiently expressed in onion epidermal cells. A magnified view of the image surrounding the nucleus is shown in (B). Expression was monitored 12h after transformation. Scale bars=100 µm. (C) Western blot analysis of OsMTP8.1 in shoot membrane fractions prepared using a discontinuous sucrose density gradient. Antibodies to marker proteins of the ER (anti-Bip), plasma membrane (anti-H^+^-ATPase), and tonoplast (anti-V-ATPase) were used to probe the blots. (D) Western blot analysis of OsMTP8.1 in microsome fractions prepared in the presence of Mg^2+^. Brix=1g of sucrose in 100g of solution.

Next, an OsMTP8.1:GFP fusion protein was transiently expressed in onion epidermal cells. The fluorescence of cells transfected with GFP alone was detected in the nucleus and cytoplasm (Fig. 4A). In contrast, the fluorescence of the fusion protein was observed at the cell periphery (Fig. 4B) but not in the nucleus (magnified view in Fig. 4B), suggesting that OsMTP8.1 localized to the tonoplast. To validate this result, the function of OsMTP8.1:GFP was investigated in yeast. Expression of *OsMTP8.1:GFP* rescued the Mn sensitivity of Δ*pmr1* similarly to *OsMTP8.1* (Supplementary Fig. S2 at *JXB* online), thereby suggesting that the fusion protein plays the same role as OsMTP8.1. To confirm these results, western blot analysis of microsomal fractions prepared using a discontinuous sucrose gradient was performed. OsMTP8.1 was detected in the same fraction as the tonoplast marker V-ATPase and the ER marker luminal binding protein (Bip) (Fig. 4C). Further, OsMTP8.1 was not detected in fractions positive for the plasma membrane marker H^+^-ATPase. To identify the fractions containing V-ATPase and Bip, we performed western blot analysis of microsomes fractionated in the presence of Mg^2+^ using a continuous sucrose gradient. The highest levels of V-ATPase and OsMTP8.1 were detected in the same fraction (Fig. 4D). In contrast, peak levels of Bip were detected in the higher density fractions. These results indicate that OsMTP8.1 is localized to the tonoplast.

### Phenotype of OsMTP8.1 mutant rice

**Fig. 5.**
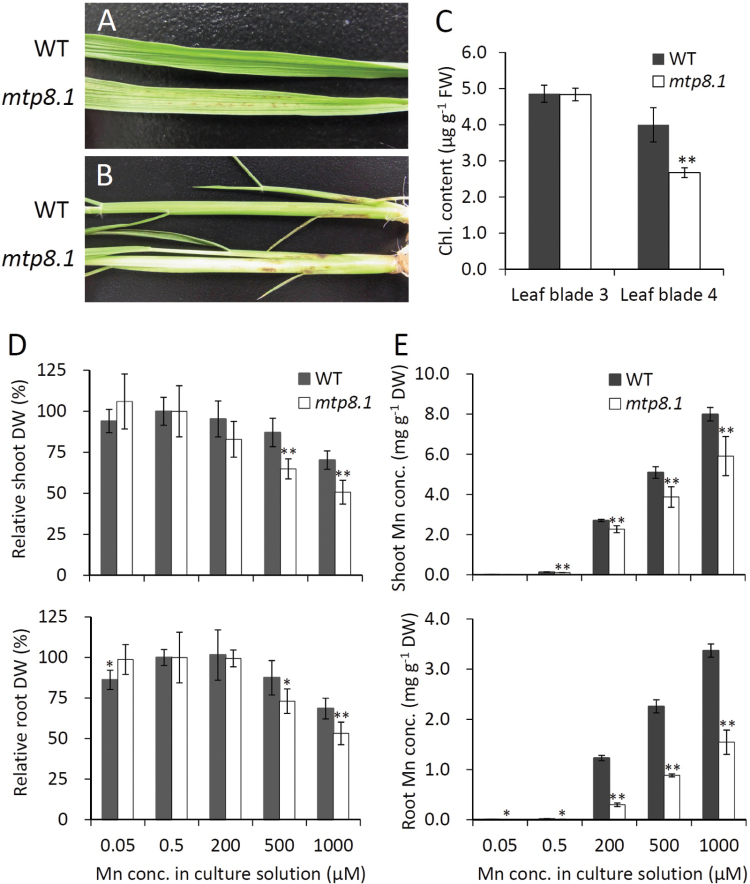
Effects of disrupting *OsMTP8.1* on Mn tolerance and accumulation. Plants were hydroponically grown for 12 d in Kimura B and then for 15 d in a solution containing various concentrations of Mn (0.05, 0.5, 200, 500, and 1000 µM). Data represent the mean ±SD (*n*=5). Significant differences between wild-type and *mtp8.1* lines were calculated using Student’s *t*-test and are indicated by ** (*P* < 0.01) and * (*P* < 0.05). (A, B) Symptoms of Mn toxicity appeared on the leaf blade (A) and sheath (B) in the presence of 500 µM Mn. (C) The SPAD (chlorophyll) values of the third and fourth leaf blades (the youngest) when plants were grown in the presence of 500 µM Mn. (D) Dry weight (DW) of shoots and roots. DW values in the presence of 0.5 µM Mn treatment were defined as 100% (control). (E) Mn concentration in shoots and roots.

**Fig. 6.**
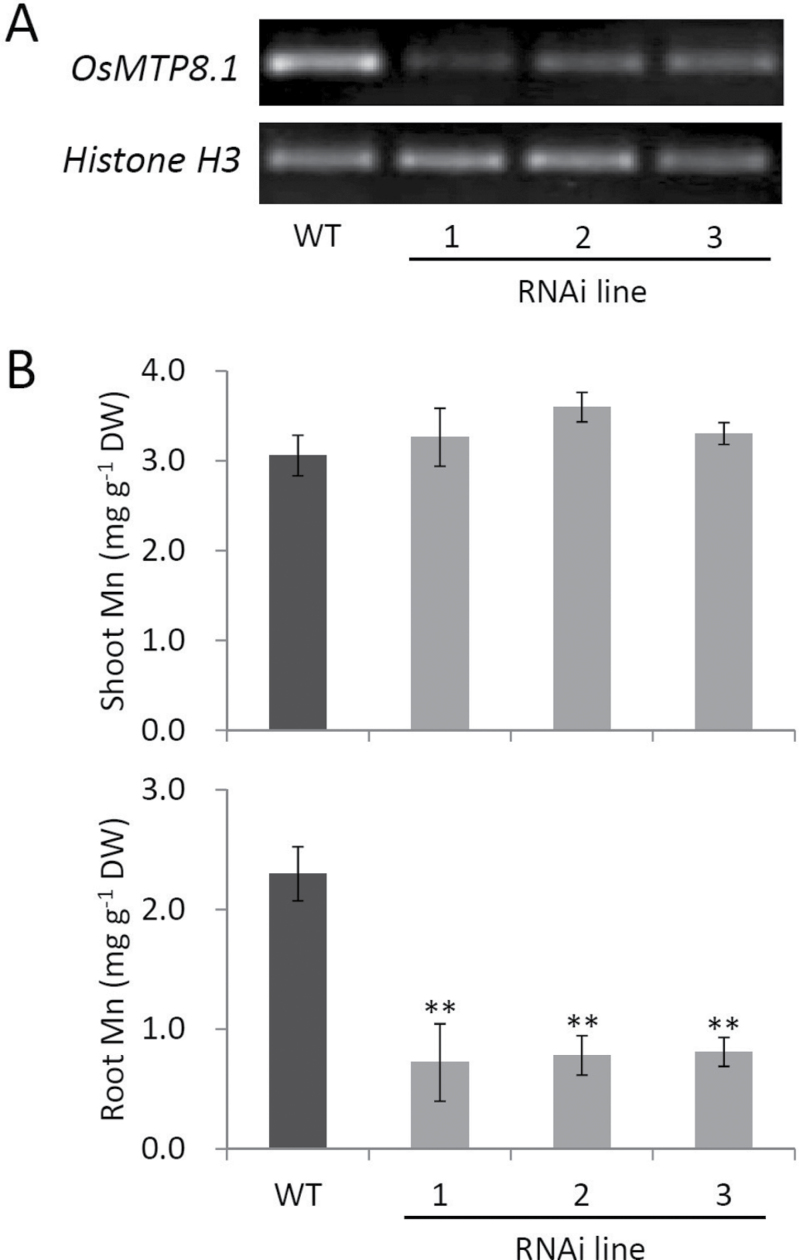
Effect of *OsMTP8.1* knockdown on Mn accumulation. (A) Semi-quantitative RT–PCR analysis of *OsMTP8.1* mRNA levels in wild-type and three independent *OsMTP8.1* RNAi lines. (B) Mn concentration in shoots and roots. Plants were hydroponically grown for 11 d in Kimura B and then for 10 d in a solution containing 200 µM Mn. Data represent the mean ±SD (*n*=3–5). Significant differences between wild-type and RNAi lines calculated using Dunnett’s test are indicated by ** (*P* < 0.01).

To investigate the role of OsMTP8.1 *in planta*, the Mn tolerance and accumulation of wild-type rice and a homozygous Tos-17 insertion line were compared. The mutant exhibited symptoms of Mn toxicity, such as brown spots and chlorosis, in the youngest fully expanded leaf blades (Fig. 5A) and sheaths (Fig. 5B) in the presence of 500 µM Mn. The chlorophyll content in the youngest leaf blades decreased (67%, *P* < 0.01), but that in the older leaf blades did not (Fig. 5C). There were no significant differences in the growth rate of the wild type and *mtp8.1* when the external Mn concentration was <200 µM; however, when the external Mn was >500 µM, *mtp8.1* growth was impaired (Fig. 5D). Compared with wild-type plants, Mn accumulation in shoots of *mtp8.1* was slightly reduced (16–26%), while that in the roots was more significantly reduced (54–76%) (*P* < 0.01, Fig. 5E). To verify the effect of functionally deleting OsMTP8.1 on Mn accumulation, *OsMTP8.1* RNAi lines were generated and compared with wild-type plants. Semi-quantitative RT–PCR showed reduced *OsMTP8.1* expression in three independent RNAi lines (Fig. 6A). Mn accumulation was significantly reduced in the roots (65–69%, *P* < 0.01) but not in the shoots of RNAi lines (Fig. 6B). Unlike Mn, there were no large differences in the accumulation of other microelements (Zn, Cu, and Fe) and macroelements (K, Mg, and Ca) in the shoots and roots between the Tos-17 insertion mutant and the wild type (Supplementary Fig. S3 at *JXB* online). Similar results were obtained with RNAi lines (Supplementary Fig. S4), suggesting that OsMTP8.1 is a specific Mn transporter.

**Fig. 7.**
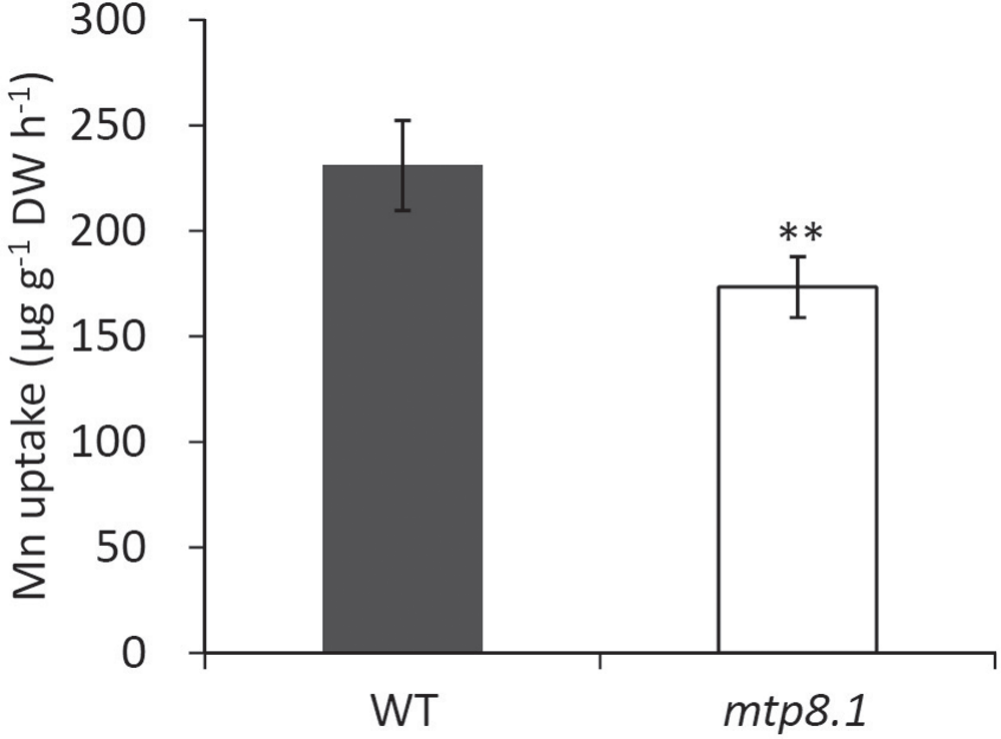
Effect of inhibiting *OsMTP8.1* expression on Mn uptake. Plants (22 d old) were exposed to uptake solution containing 200 µM Mn for 16h. Data represent the mean ±SD (*n*=5). Significant differences between wild-type and *mtp8.1* lines calculated using Student’s *t*-test are indicated by ** (*P* < 0.01).

To determine why inhibiting the expression of *OsMTP8.1*, which is specifically localized in the shoots, resulted in low Mn accumulation in the roots, particularly in the presence of high Mn concentrations, Mn uptake by the roots of knockout lines was compared with that of the wild-type plants, and it was found that Mn uptake by *mtp8.1* was 75% of the wild-type level within 16h (*P* < 0.01, Fig. 7). The expression of *OsNramp5*, which encodes a major Mn transporter, was also analysed. However, there was no significant difference in the expression levels of this gene between the wild type and *mtp8.1* (Supplementary Fig. S5 at *JXB* online).

## Discussion

### OsMTP8.1 is involved in Mn detoxification in young leaves

In the present study, using cDNA expression library cloning techniques, *OsMTP8.1*, which encodes a member of the five Mn-CDFs in rice was isolated (Fig. 1). OsMTP8.1 is classified into Group 8 (Gustin *et al*., 2011). In this subgroup, *ShMTP8* was first cloned from the tropical legume *S. hamata* using the same approach as in this study (Delhaize *et al*., 2003). ShMTP8 is implicated in transport of Mn into vacuoles, and heterologous expression of this gene conferred Mn tolerance in *A. thaliana* and *S. cerevisiae*. However, the role of this protein in plants is not clear because loss-of-function studies have not been conducted owing to technical difficulties. The closest *OsMTP8.1* orthologue in *A. thaliana*, *AtMTP8*, shows extremely low transcription levels compared with that of other Mn-CDFs (Delhaize *et al*., 2007), and the function of the encoded protein is unclear. To the authors’ knowledge, OsMTP8.1 is the first characterized transporter in Group 8 Mn-CDFs *in planta*.

Heterologous expression of *OsMTP8.1* in *S. cerevisiae* resulted in enhanced tolerance and accumulation of Mn, but not in other heavy metals (Fig. 2). Knockout and knockdown of *OsMTP8.1* decreased Mn accumulation without changing other metal concentrations (Figs 5E, 6B; Supplementary Figs S3, S4 at *JXB* online). Although no direct evidence could be obtained indicating Mn transport activity, the results suggest that OsMTP8.1 could be a specific transporter for Mn. Accumulation of OsMTP8.1 increases following exposure to elevated levels of Mn (Fig. 3C); however, even a 4000-fold difference in Mn supply (0.05 µM versus 200 µM) induced only a 1.6-fold difference in the protein level, indicating that OsMTP8.1 accumulation is substantially constitutive. Since rice grows in flooded paddy fields with very high Mn availability, the constitutive trait may be necessary for continuous Mn detoxification.

*OsMTP8.1* was expressed in all cells of leaf blades and localized to the tonoplast (Fig. 3, 4). Disruption of *OsMTP8.1* exacerbated Mn toxicity, which is characterized by the presence of brown spots and chlorosis in young leaves and inhibited growth (Fig. 5). These results suggest that OsMTP8.1 plays a role in Mn detoxification by sequestering Mn into vacuoles in shoots. Previous studies indicate that high Mn tolerance in rice leaves is associated with enhanced binding of Mn to a chloroplast-localized protein, but not accumulation in vacuoles (Lidon and Teixeira, 2000; Lidon, 2001; Lidon *et al*., 2004). Thus, the present findings indicate a mechanism of Mn tolerance that involves the sequestration of Mn into the vacuoles in rice shoot. An Mn-nicotianamine transporter OsYSL6 mediates Mn tolerance by lowering the concentration of Mn in the apoplastic solution (Sasaki *et al*., 2011). Disruption of OsYSL6 causes necrosis in the oldest leaf blades, which contained the highest concentration of Mn in the shoot. This suggests that OsYSL6 has a role distinct from that of OsMTP8.1 which is required for Mn detoxification in young leaves.

### Possible role of OsMTP8.1 in regulation of root Mn uptake

Interestingly, inhibition of *OsMTP8.1* expression by knockout or knockdown techniques resulted in significantly reduced accumulation of Mn in the roots (Figs 5E, 6B), although *OsMTP8.1* was mainly expressed in shoots (Fig. 3). Rice plants efficiently transfer Mn to the shoots rather than retaining it in the roots. Because of the low Mn concentration in the roots, the residual OsMTP8.1 may be sufficient to sequester Mn into vacuoles to some extent, and disruption of the storage function may reduce the Mn concentration in *osmtp8.1*. Alternatively, the reduced Mn accumulation can be attributed to the reduction in uptake.

Plants employ various mechanisms to ensure appropriate uptake, distribution, and detoxification of heavy metals. For example, the higher Mn tolerance of subterranean clover in comparison with that of toothed medick was attributed to a lower uptake rate and greater retention in the root (Robson *et al*., 1970). In rice, it is speculated that Mn toxicity may be associated with the efficiency of Mn uptake. It is assumed that the disruption of *OsMTP8.1* resulted in retention of Mn in the cytosol, which in turn stimulated the response to Mn toxicity by inhibiting further uptake of Mn, and the suppression of Mn uptake ultimately decreased Mn accumulation. Moreover, the results show that the uptake rate in *osmtp8.1* is lower than that in the wild type (Fig. 7), which supports this hypothesis. Sasaki *et al*. (2012) recently identified OsNramp5 as a major transporter involved in the uptake of Mn and Cd from the rhizosphere to root cells. Knockout of this gene resulted in decreased Mn accumulation in both shoots and roots. Therefore, expression of *OsNramp5* in the wild type and *osmtp8.1* cultivated in the presence of high Mn concentrations was compared. However, there was no difference in *OsNramp5* expression between the wild-type rice and the knockout line (Supplementary Fig. S5 at *JXB* online). Accumulation of OsNramp5 could be controlled post-transcriptionally, although the authors are not aware of studies that prove this. Thus, the molecular mechanism responsible for decreased Mn concentration in the knockout line remains to be determined; however, the present results suggest that several transporters involved in Mn uptake in rice remain undiscovered.

In conclusion, it is shown that OsMTP8.1 localizes to the tonoplast and plays an important role in Mn homeostasis, presumably by sequestering Mn specifically into vacuoles in the cells of rice shoots.

## Supplementary data

Supplementary data are available at *JXB* online.

Figure S1. Analysis of the Tos-17 insertion mutant.

Figure S2. Effect of *OsMTP8.1:GFP* expression on Mn tolerance in *Saccharomyces cerevisiae.*

Figure S3. Effect of *OsMTP8.1* knockout on the accumulation of microelements (Fe, Zn, and Cu) and macroelements (K, Mg, and Ca).

Figure S4. Effect of *OsMTP8.1* knockdown on accumulation of Fe, Zn, and Cu.

Figure S5. Quantitative real-time RT–PCR analysis of *OsNramp5* transcription in roots.

Supplementary Data
