# Supplementary material for: Mn tolerance in rice is mediated by MTP8.1, a member of the cation diffusion facilitator family
Source: J Exp Bot. 2013 Aug 20;64(14):4375–87. doi: 10.1093/jxb/ert243 (PMC3808320; doi:10.1093/jxb/ert243)
Supplement: Supplementary Data [file supp_64_14_4375__index.html]

Mn tolerance in rice is mediated by MTP8.1, a member of the cation diffusion facilitator family — Mn tolerance in rice is mediated by MTP8.1, a member of the cation diffusion facilitator family — Supplementary Data 

# Mn tolerance in rice is mediated by MTP8.1, a member of the cation diffusion facilitator family

## 

Data files

**Files in this Data Supplement:**

- Supplementary Data - Supplementary Data
