# Supplementary material for: Mn tolerance in rice is mediated by MTP8.1, a member of the cation diffusion facilitator family
Source: J Exp Bot. 2013 Aug 20;64(14):4375–87. doi: 10.1093/jxb/ert243 (PMC3808320; doi:10.1093/jxb/ert243)
Supplement: Supplementary Data [file supp_ert243_jexbot098442_file001.pdf]

Mn tolerance in rice is mediated by OsMTP8.1, a member of the cation diffusion facilitator family

Zonghui Chen, Yumi Fujii, Naoki Yamaji, Sakine Masuda, Yuma Takemoto, Takehiro Kamiya, Yusufujiang Yusuyin, Kozo Iwasaki, Shin-ichiro Kato, Masayoshi Maeshima, Jian Feng Ma, Daisei Ueno

Supplementary materials

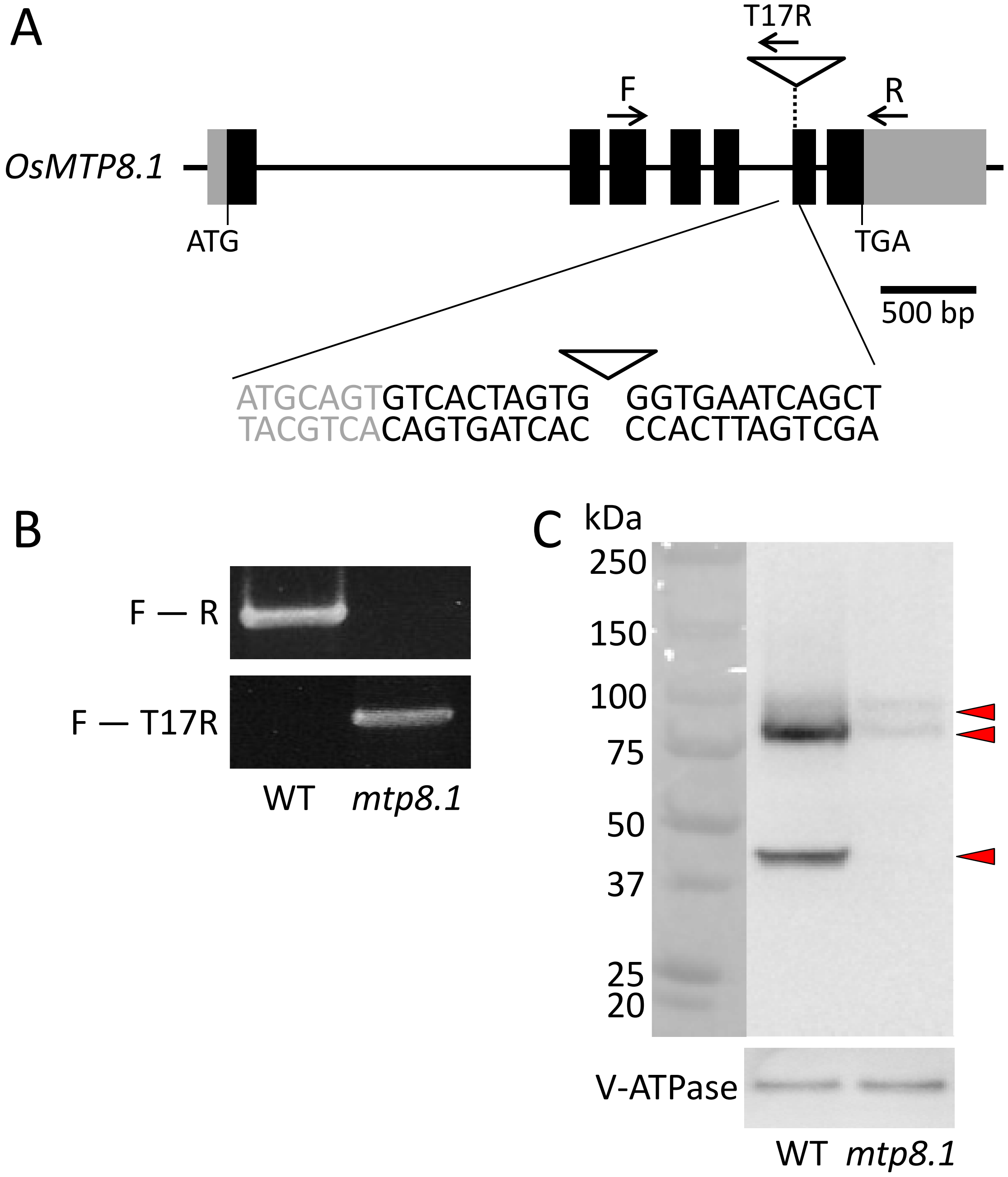

Supplementary Figure S1. Analysis of Tos-17 insertion mutant

(A) Localization of Tos-17 insertion in osmtp8.1 mutant allele. Grey boxes, black boxes, and black lines indicate untranslated region, exon of coding region, and intron, respectively. Tos-17 is shown to be located on exon 6 by a triangle. Primers designed for *OsMTP8.1* (F and R) and Tos-17 (T17R) for genotyping are shown by horizontal arrows. The base sequence highlights around the site of Tos-17 insertion. Grey and black letters show intron and exon, respectively.

(B) Genotype of wild-type and *mtp8.1*. The PCR products amplified from genomic DNA using primers F-R or F-T17R are indicated.

(C) Western blot analysis of *OsMTP8.1* in microsomal membranes prepared from shoots of wild-type and mtp8.1 rice lines. The tonoplast marker protein V-ATPase was detected using a specific antibody. The arrowhead indicates predicted OsMTP8.1.

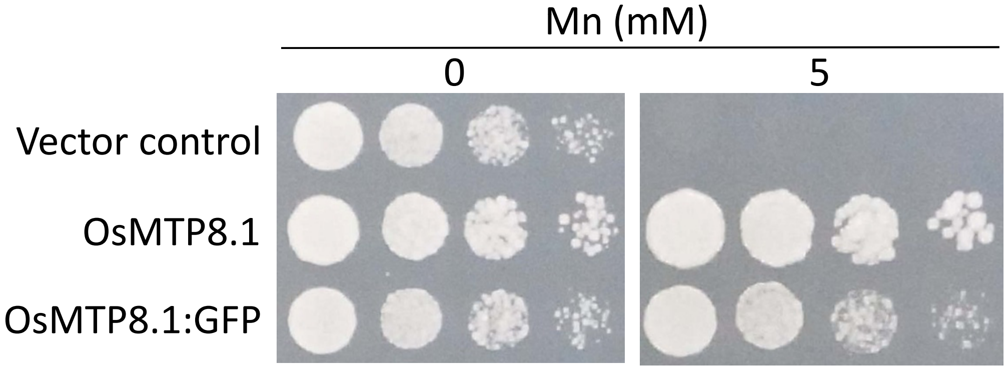

Supplementary Figure S2. Effect of *OsMTP8.1:GFP* expression on Mn tolerance in *S. cerevisiae*. The yeast mutant Δ*pmr1* carrying the pYES2 empty vector, pYES2-*OsMTP8.1* or pYES2-*OsMTP8.1:GFP* were used. Five µl (OD_{600} = 2.0) of serial dilutions (10-fold) were spotted onto SC-U/Gal medium with or without (control) supplementation with 5 mM $MnCl_2$. Plates were incubated for 48 h at 30 ºC in the dark.

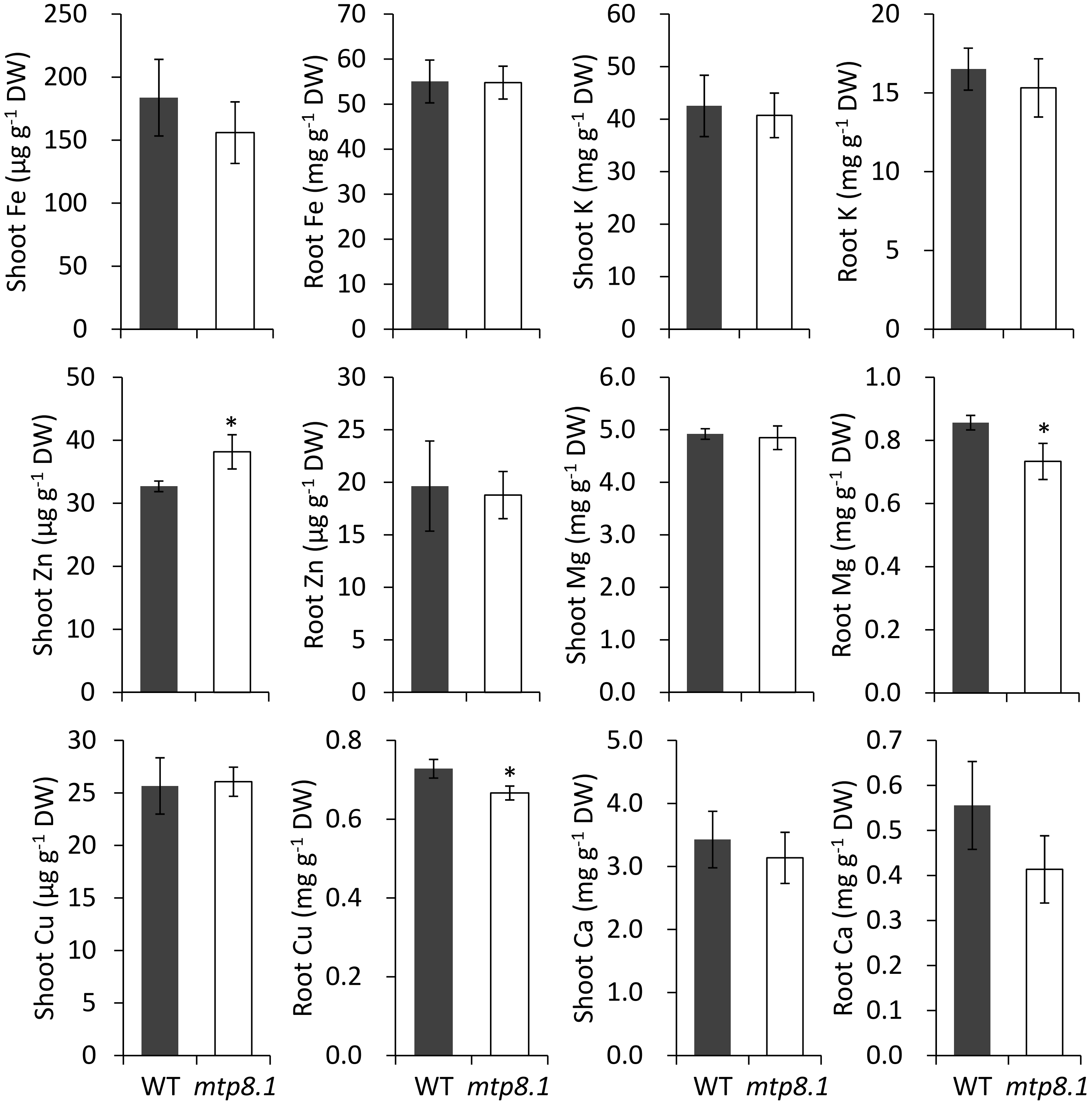

Supplementary Figure S3. Effect of *OsMTP8.1* knockout on the accumulation of microelements (Fe, Zn and Cu) and macroelements (K, Mg and Ca). Plants were hydroponically grown for 11 d in Kimura B and then for 10 d in a solution containing 200 µM Mn. Data represent the mean ± standard deviation (SD) (n = 4). Significant differences between wild-type and *mtp8.1* were determined using Student's *t*-test are indicated by * ($P < 0.05$)

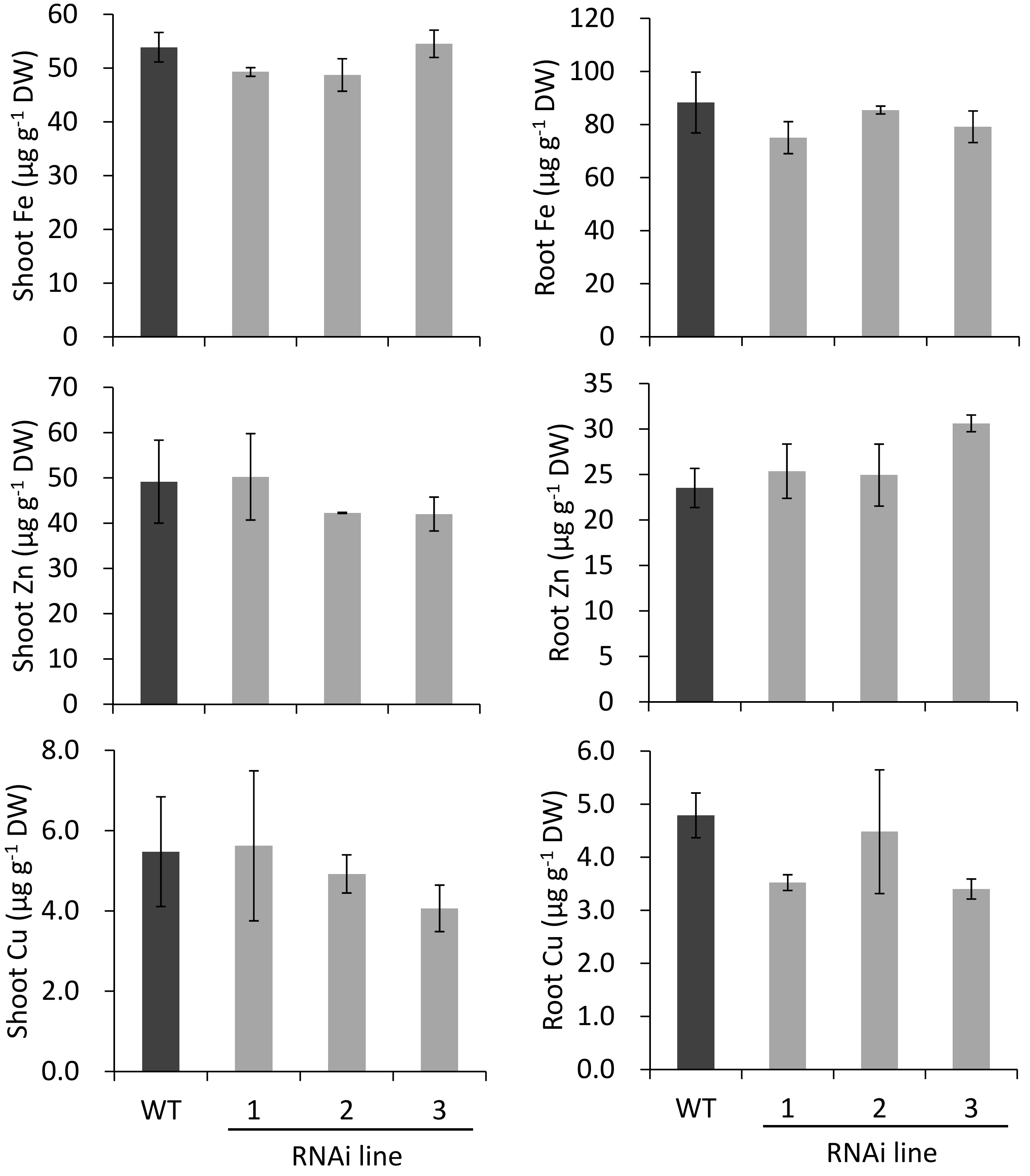

Supplementary Figure S4. Effect of *OsMTP8.1* knockdown in accumulation of Fe, Zn and Cu. Plants were hydroponically grown for 11 d in Kimura B and then for 10 d in a solution containing 200 μM Mn. Data represent the mean ± SD (*n* = 3-5). Significant differences between wild-type and RNAi lines were calculated using Dunnett's test.

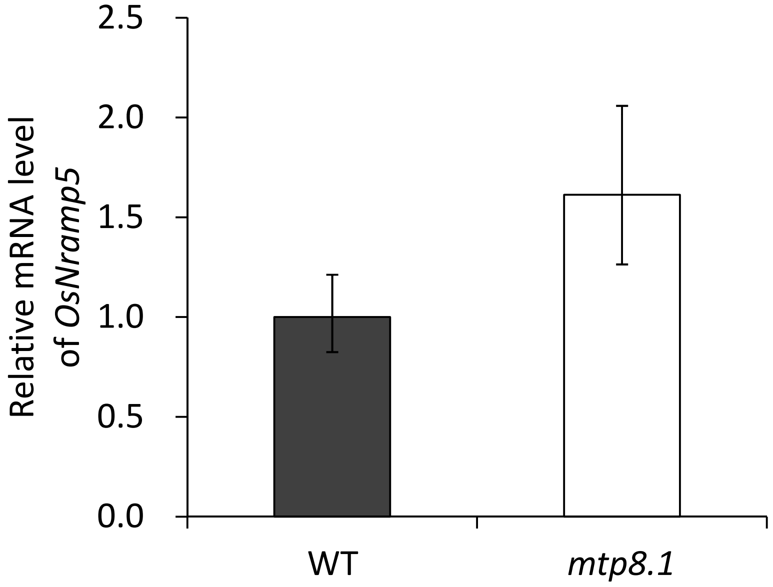

Supplementary Figure S5. Quantitative real time RT-PCR analysis of *OsNramp5* transcription in roots. Plants were hydroponically grown for 21 d and then for 12 d in a solution containing 200 μM Mn. *Histone H3* was used as an internal control. Expression relative to wild-type is shown. Data represent the mean ± SD (n = 4).
